# An immune-related exosome signature predicts the prognosis and immunotherapy response in ovarian cancer

**DOI:** 10.1186/s12905-024-02881-y

**Published:** 2024-01-18

**Authors:** Kaibo Zhu, Jiao Ma, Yiping Tian, Qin Liu, Jun Zhang

**Affiliations:** 1 Department of Pathology, Women’s Hospital School of Medicine Zhejiang University, No.3, East Qingchun Road, Shangcheng District, Hangzhou, China; 2 Department of Pathology, Zhejiang Hospital, Hangzhou, China; 3 Department of Pathology, Zhejiang Cancer Hospital, Hangzhou, China; 4 Department of Clinical Laboratory, Sir Run Run Shaw Hospital Zhejiang University School of Medicine, No.1, Xueshi Road, Shangcheng District, Hangzhou, China

**Keywords:** Biomarker, Immune-related Exosome, Immunotherapy, Ovarian Cancer, Prognosis, Tumor Microenvironment

## Abstract

**Background:**

Cancer-derived exosomes contribute significantly in intracellular communication, particularly during tumorigenesis. Here, we aimed to identify two immune-related ovarian cancer-derived exosomes (IOCEs) subgroups in ovarian cancer (OC) and establish a prognostic model for OC patients based on immune-related IOCEs.

**Methods:**

The Cancer Genome Atlas (TCGA) database was used to obtain RNA-seq data, as well as clinical and prognostic information. Consensus clustering analysis was performed to identify two IOCEs-associated subgroups. Kaplan-Meier analysis was used to compare the overall survival (OS) between IOCEs-high and IOCEs-low subtype. Gene Ontology (GO) and Kyoto Encyclopedia of Genes and Genomes (KEGG) analyses were conducted to investigate the mechanisms and biological effects of differentially expressed genes (DEGs) between the two subtypes. Besides, an IOCE-related prognostic model of OC was constructed by Lasso regression analysis, and the signature was validated using GSE140082 as the validation set.

**Results:**

In total, we obtained 21 differentially expressed IOCEs in OC, and identified two IOCE-associated subgroups by consensus clustering. IOCE-low subgroup showed a favorable prognosis while IOCE-high subgroup had a higher level of immune cell infiltration and immune response. GSEA showed that pathways in cancer and immune response were mainly enriched in IOCE-high subgroup. Thus, IOCE-high subgroup may benefit more in immunotherapy treatment. In addition, we constructed a risk model based on nine IOCE-associated genes (CLDN4, AKT2, CSPG5, ALDOC, LTA4H, PSMA2, PSMA5, TCIRG1, ANO6).

**Conclusion:**

We developed a novel stratification system for OV based on IOCE signature, which could be used to estimate the prognosis as well as immunotherapy for OC patient.

**Supplementary Information:**

The online version contains supplementary material available at 10.1186/s12905-024-02881-y.

## Background

Ovarian cancer (OC) is one of the most deadly gynecologic malignancy [1], ranking the fifth main cause of cancer death in female [2]. Due to the asymptomatic characteristic and inadequate screening tests in OC [3], this cancer is usually diagnosed at advanced stages and thus has a poor prognosis with five-year survival rates under 45% [4]. Depending on the morphological type, OC is classified into several subtypes: serous carcinomas, mucinous carcinomas, endometrioid carcinomas, clear-cell carcinomas, mixed, and undifferentiated type [5]. Despite the multiple subtypes of OC, they are considered as a single entity [6]. OC can be diagnosed initially with transvaginal ultrasonography and serum CA125 measurement, but both of these tests are not specific for detecting the disease. The prognosis of OC mainly depends on the cancer’s stage and grade due to the lack of reliable prognostic markers. Until now, there have been a wide variety of clinical trials focus on immune checkpoint inhibitors [7–9], however, there have been no significant improvements of the clinical cure rate for a limited response to immunotherapy in OC. Thus, it is imperative to develop effective biomarkers to predict the prognosis and stratify patients that may improve the predictiveness of response.

Exosomes are approximately 100 nm extracellular vesicles that exist in most body fluids, which are secreted and released by a variety of cells into the extracellular matrix. Exosomes play an important role in various biological and pathological processes by transporting bioactive materials such as DNAs, proteins, messenger RNAs (mRNAs), non-coding RNAs or micro RNAs (miRNAs) into the target cells [10–12]. In terms of practice, exosomes have shown their value in early diagnosis and management of endometriosis through liquid biopsy [13]. Recent studies have reported that exosomes can promote tumor development [14], induce drug resistance in cancers [15], modulate tumor immune response or activate anti-tumor immune response [16–19]. It is generally known that cancer cells secret larger quantities of exosomes than normal cells, and those cancer-derived exosomes show robust ability to affect the microenvironment of both local and distant sites [20]. Ovarian cancer-derived exosomes (OCE) exist in a variety of body fluids including urine, ascites, and blood serum. Recent studies reported that OCE could accelerate ovarian cancer development via modulating the microenvironment [21, 22], transform fibroblasts into cancer associated fibroblasts (CAFs) [23, 24], suppress immune cells and promote macrophages to become tumor-associated macrophages [25, 26], and increase ovarian cancer infiltration by inducing apoptosis of peritoneal mesenchymal cells [27, 28]. A panel of extracellular vesicles-related miRNAs and proteins may act in future as a “cancer signature”, representing a useful new weapon in OC screening, diagnosis and prognostic assessment [29]. Thus, it would prove valuable to identify biomarkers that classify patients based on their response to IOCE immunotherapy.

In the current study, we first identify IOCE biomarkers involved in the prognosis, immune microenvironment and immune response of OC. In addition, we constructed an IOCE risk model that predicts the prognosis in OC and further an independent Gene Expression Omnibus (GEO) dataset was used as the validation set. This study would be a robust supplement in the ovarian cancer immunotherapy field.

## Methods

### Datasets acquisition and data processing

TCGA ovarian cancer dataset (*n* = 376) and Genotype-Tissue Expression Portal (GTEX) dataset (*n* = 180) were utilized to download gene expression data of ovarian cancer and normal samples, corresponding survival and clinical information. Further, we utilized the GSE140082 dataset (https://www.ncbi.nlm.nih.gov/geo/, accessed on 20 October 2022) as the validation set to verify IOCE risk model that predicts the prognosis in OC. The ovarian cancer-derived exosomes were obtained from the ExoCarta database (http://www.exocarta.org/, accessed on 21 October 2022). In addition, immune-related genes were download from the Gene List module of the Immunology Database and Analysis Portal (ImmPort) database.

### Acquisition of IOCEs

First, we identified the DEGs between the OC samples and normal samples in the TCGA dataset using the “limma” package. The screening criteria was log2-fold change ≥ 1.5 and adjusted *P* value < 0.05. Then, an intersection among the DEGs, the exosome-encapsulated genes, immune-related genes progno-sis-related genes was made to acquire 21 IOCEs. Venn diagrams and the R package “heatmap” were used to visualize the results.

### Analysis of consensus clustering

Consensus clustering analysis was performed by using the ConsensusClusterPlus tool in R to classify molecular subtypes associated with IOCEs. After that, the optimal cluster number between k = 2–10 was assessed and a 1000-time replication was conducted to ensure that the results would be stable. A cluster map was created by using the pheatmap tool in R.

### Screening and functional enrichment analysis of DEGs

The differentially expressed genes between IOCE low and high subgroup were screened by the Limma package in R software. An adjusted *P* value < 0.05 and |fold change| >2 was set as the screening criteria. Gene Ontology (GO) and Kyoto Encyclopedia of Genes and Genomes (KEGG) [30] analyses were performed to analyze the biological effects and pathway of the DEGs.

### Characterization of signaling mechanisms between two IOCE cohorts

To identify biological functions enriched between the IOCE low and high subgroups, we performed GSEA by GSEA v4.0 (https://www.gsea-msigdb.org/, accessed on 26 October 2022), with C2 (c2.cp.kegg. v7.1.symbols.gmt) curated gene sets from the Molecular Signatures Database (MSigDB). The results with *P* < 0.05 and FDR < 0.25 were considered significantly enriched after 1000 permutations per analysis.

### Analysis of somatic mutation

TCGA GDC Data Portal was used to obtain the somatic mutation data for the OC samples. To visualize the mutated genes between the IOCE low and high subgroups, waterfall plots were performed using the “Maftools” package in R software.

### Immune response between two IOCE cohorts

Expression data of OC samples were loaded into CIBERSORT (https://cibersort.stanford.edu/, accessed on 26 October 2022) and repeated 1000 times to identify immune characteristics of 376 OC patients. Next, the relative percentage of 22 immune cell types between the IOCE low and high subgroups were compared, and a landscape map was utilized to visualize the results.

### Construction and verification of the IOCE-associated risk model

In total, we identified 21 IOCEs and a nine IOCE-related genes risk model was constructed by the Lasso regression analysis. Eventually, the corresponding nine IOCEs were selected into the model, which was CLDN4, AKT2, CSPG5, ALDOC, LTA4H, PSMA2, PSMA5, TCIRG1, and ANO6. Furthermore, the corresponding regression coefficients were calculated, which were 0.0839, 0.0718, -0.0762, 0.0594, 0.0276, -0.1654,-0.1723, 0.0248, and 0.009, respectively. By using the formula above in combination with the beta value of multivariate Cox regression, the risk score formula is as follows: Riskscore=(-0.0839)×CLDN4+(0.0718)×AKT2+(-0.0762)×CSPG5+(0.0594)×ALDOC+(0.0276)×LTA4H+(-0.1654)×PSMA2+(-0.1723)×PSMA5+(0.0248)×TCIRG1+(0.009)×ANO6. Kaplan-Meier analysis was performed to compare the overall survival (OS) between the IOCE low and high subgroups. Subsequently, both univariate and multivariate Cox regression analyses were performed, taking into account common factors associated with OC. In addition, we selected the GSE140082 as the validation set to validate the IOCE-related genes risk model established based on TCGA database.

### Expression of IOCE-related genes expression

To verify the protein expression of IOCE-related genes expression in normal and tumor tissues, data from Human Protein Atlas (HPA) were downloaded (https://www.proteinatlas.org/, accessed on 25 October 2022) and determined whether the differences in protein expression levels were consistent with the previous mRNA expression from TCGA.

## Results

### Identification and pathway enrichment analysis of DEGs in normal and OC samples

**Fig. 1:**
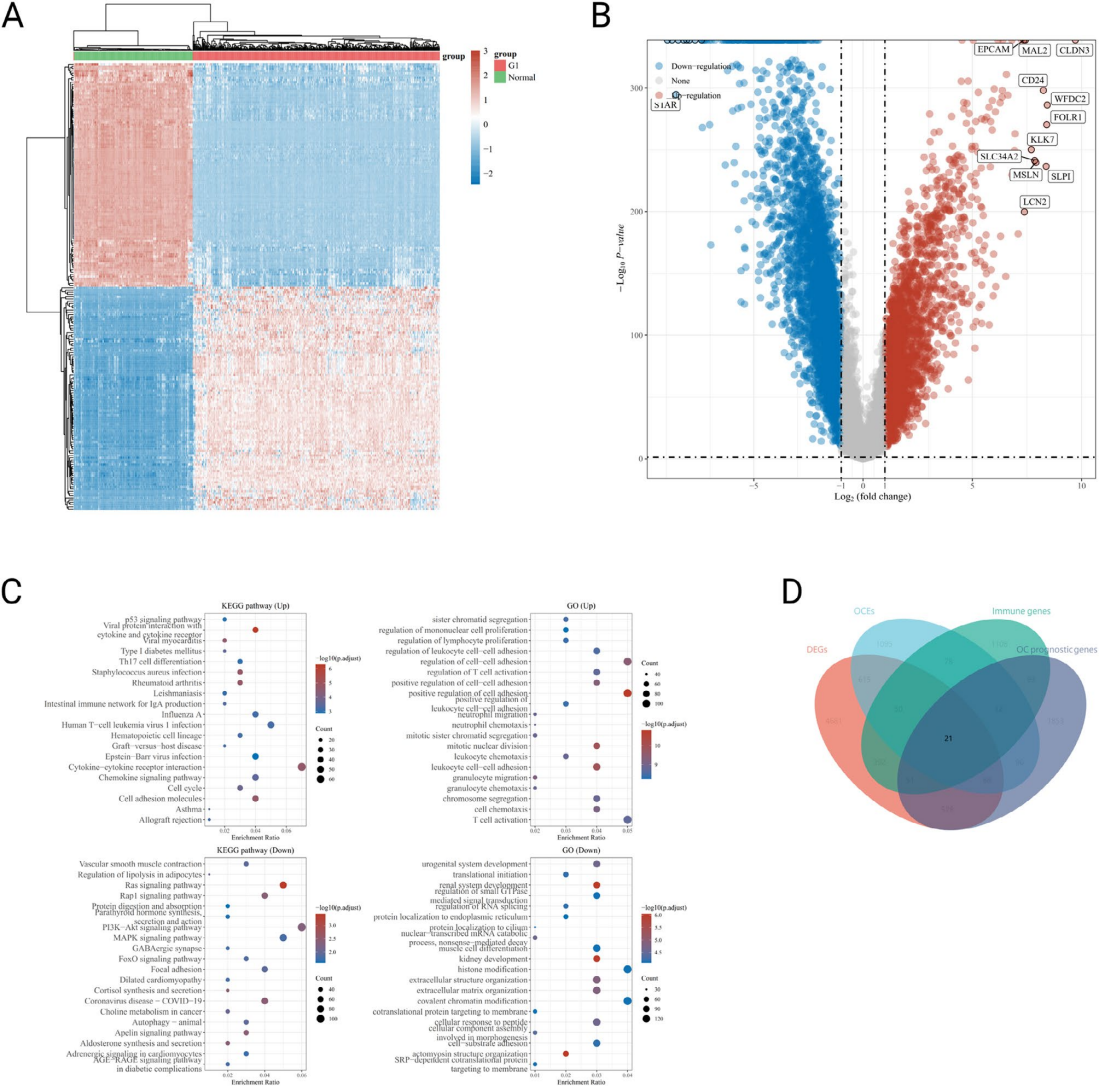
Identification of IOCE-associated genes by differential analysis: (**A**) Heatmap of DEGs between OC and normal samples; (**B**) Volcano plot of 6406 DEGs; (**C**) GO and KEGG analysis of DEGs between OC and normal samples; (**D**) Venn diagram of the intersection of DEGs, exosome-encapsulated genes, prognosis-related genes and immune-related genes. Note: DEGs: differentially expressed genes

In total, 376 OC patients from TCGA database and 180 normal samples from GTEX were included in the training set, and a differential analysis were performed to identify DEGs in OC and normal samples. A total of 6406 DEGs were identified, among which 2333 DEGs were up-regulated while 4073 DEGs were down-regulated, visualized with the R package “heatmap” and the volcano maps (Fig. 1A-B). Go and KEGG analysis were then performed to investigate signal pathway and biological effects of DEGs. Go analysis showed the up-regulated genes were mainly involved in immune process, for instance regulation of mononuclear cell proliferation, regulation of lymphocyte proliferation, regulation of cell–cell adhesion and so on. KEGG enrichment analysis showed the up-regulated genes were enriched in a variety of immune-associated pathways, such as Th17 cell differentiation, Cytokine–cytokine receptor interaction and Human T-cell leu-kemia virus 1 infection (Fig. 1C). The results indicated the up-regulated DEGs were tightly associated with the immunity.

Then, an intersection between the 6406 DEGs, the 2035 exosome-encapsulated genes download from the ExoCarta database (Supplementary Table 1), 2727 prognosis-related genes in OC identified by univariate Cox regression analysis (Supplementary Table 2) and 2483 immune-related genes from the ImmPort database (Supplementary Table 3) was made to obtain 21 immune-related ovarian cancer-derived exo-somes (IOCEs). Venn diagrams were used to visualize the results (Fig. 1D).

### Consensus clustering based on IOCEs

**Fig. 2:**
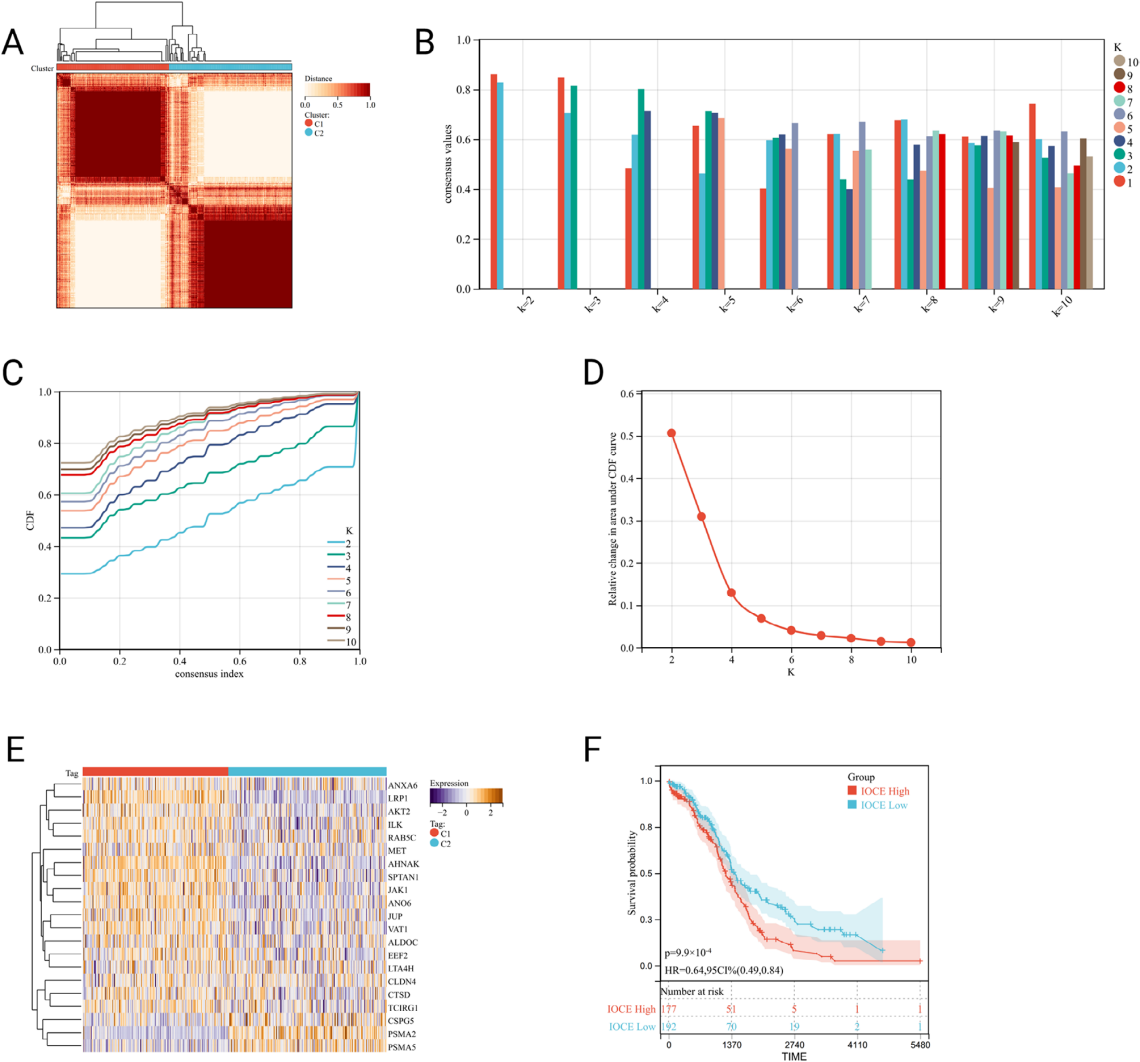
Identification of IOCE-associated subtypes by consensus clustering: (**A**) Heatmap depicts consensus clustering solution (k = 2) for 21 genes in 376 OC samples; (**B**) Consensus value of con-sensus clustering for k = 2 to 10; (**C, D**) Delta area curve of consensus clustering indicates the relative change in area under the cumulative distribution function (CDF) curve for k = 2 to 10; (**E**) Heatmap of 21 IOCE-related gene expressions in different subtypes (yellow represents high ex-pression and blue represents low expression); (**F**) Kaplan-Meier curves of OS in IOCE-high and low subtypes

The IOCE-related clusters of OC were formed using consensus clustering analysis. Based on k-means clustering, the TCGA-OC cohort was divided into two clusters corresponding the IOCE genes expression (Fig. 2A-D). In summary, there was a high level of expression of IOCE related genes in cluster C1, suggesting that the cluster was regarded as the IOCE-high subgroup. On the contrary, clusters C2 showed low expression levels of IOCE related genes indicating an IOCE-low subgroup (Fig. 2E). As a result, cluster C1 was defined as IOCE-high subgroup, and cluster C2 as IOCE-low subgroup. Next, survival analysis was performed to explore the different clinical outcome in the two IOCE-based subtypes. The results showed that the IOCE-high subgroup had a poor prognosis while the IOCE-low subgroup presented a favorable clinical outcome (Fig. 2F).

### Diverse signal pathways and DEGs involved in different IOCE subgroups

**Fig. 3:**
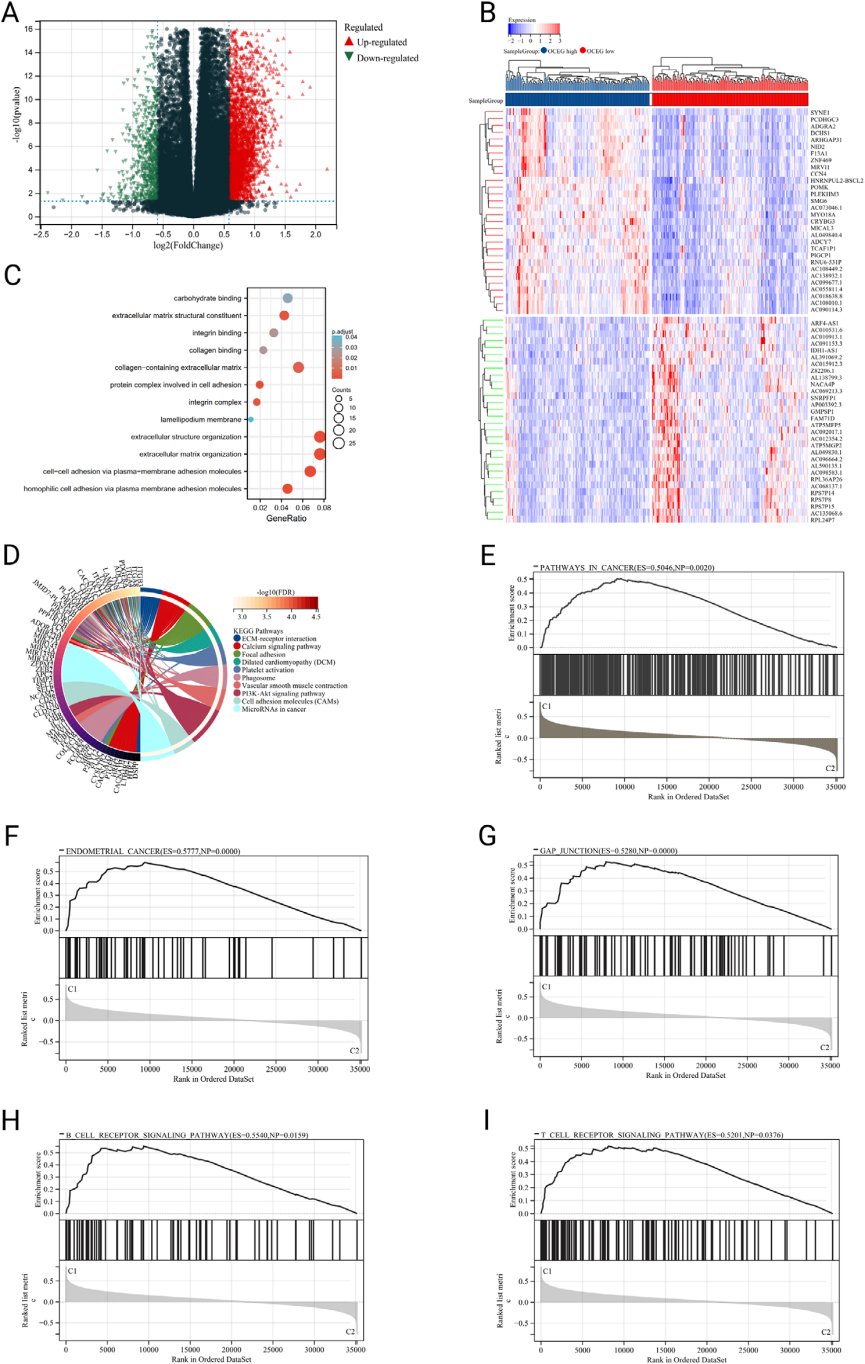
Identification of DEGs and underlying signal pathways in different subtypes: (**A**) Volcano plot presents the distribution of DEGs quantified between IOCE-high and low subtypes with threshold of |log2 Fold change|>1.5 and *P* < 0.05 in TCGA cohort; (**B**) Heatmap shows the DEG expression in different subtypes; (**C, D**) KEGG and GO signaling pathway enrichment analysis, the size of the dot represents gene count, and the color of the dot represents -log10 (p adjust-value); (**E-I**) GSEA analysis determines the underlying signal pathway between IOCE-high and low subtypes

Due to the different clinical outcome in the two IOCE-based subtypes, the key DEGs and signal pathways in each subtype need to be identified in order to understand the underlying molecular mechanism. In total, 1326 DEGs were identified including 212 up-regulated genes and 1114 down-regulated genes(Fig. 3A-B), and the DEGs in the two IOCE subgroups were enriched in carbohydrate binding, extracellular matrix structural constituent, collagen-containing extracellular matrix, protein complex involved in cell adhesion, cell–cell adhesion via plasma-membrane adhesion molecules and so on. KEGG results indicated the DEGs were involved in ECM-receptor interaction, Focal adhesion, PI3K-Akt signaling pathway, and Cell adhesion molecules (Fig. 3C-D). To further illustrate the underlying signaling pathways involved in the IOCE high subgroup, we performed GSEA to show the enriched pathways of the IOCE high and low subgroups. Results showed pathways in cancer, gap junction, B cell receptor signaling pathway and T cell receptor signaling pathway were enriched in the IOCE-high subgroup (Fig. 3E-I).

### Comparison of somatic mutations and tumor immune microenvironment in IOCE-high and low subgroups

**Fig. 4:**
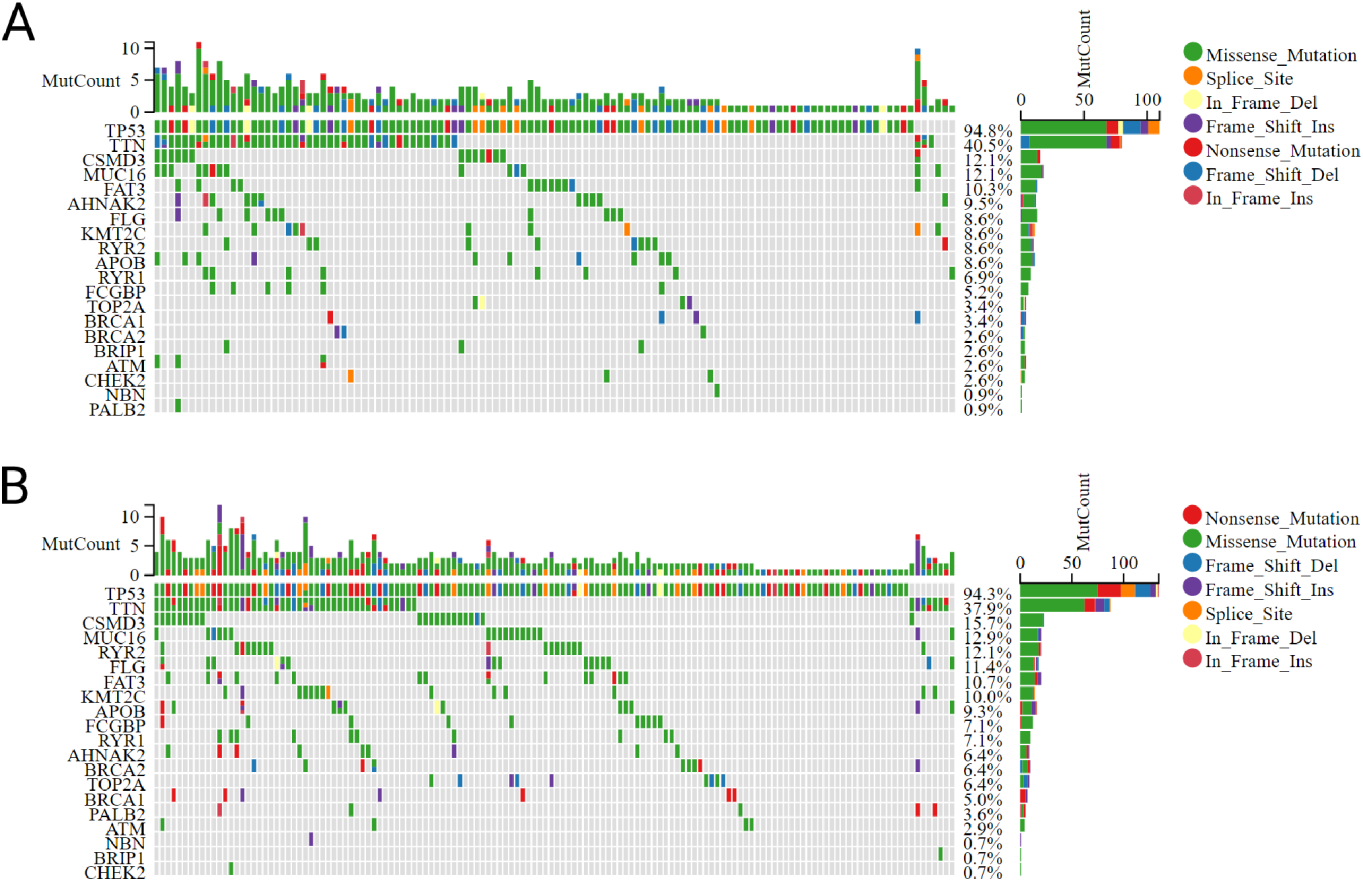
Comparison of somatic mutations between different IOCE subtypes: (**A**) Oncoprint visualization of the most frequently mutated genes in IOCE high subtype; (**B**) Oncoprint visualization of the most frequently mutated genes in IOCE low subtype

**Fig. 5:**
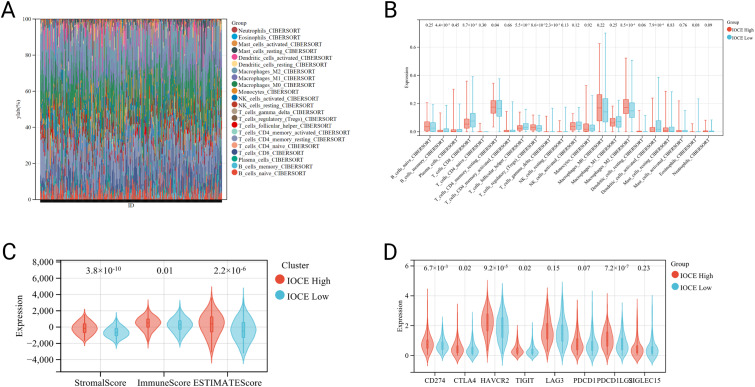
Immune landscape of IOCE-high and low subtypes: (**A**) Relative proportion of immune infiltration in IOCE-high and low subtypes; (**B**) Box plots visualize significantly different immune cells between different subtypes; (**C**) Violin plots show the median, and quartile estimations for each stromal score, immune score and ESTIMATE score; (**D**) Box plots present differential expression of multiple immune checkpoints between IOCE-high and low subtypes

Somatic mutations between the two subgroups were compared (Fig. 4A-B). Results showed mutations of TP53 and TTN in IOCE-high subgroup is higher frequency than that in IOCE-low subgroup (94.8%, 40.5% vs. 94.3%, 37.9%). Then, 22 kinds of immune cells infiltration between IOCE-high and low subgroup were compared using the CIBERSORT (Fig. 5A). Specifically, IOCE-high subgroup presented apparently increased percentages of resting CD4 + T cell memory, T cell regulatory and macrophage M2 while descend percentages of B cell memory, CD8 + T cell memory and activated dendritic cells compared to IOCE-low subgroup (Fig. 5B). To explore whether IOCE influenced the immune microenvironment in OC, we assessed the composition of the tumor microenvironment between two subgroups. In general, IOCE-high subgroup had a higher stromal score, immune score and Estimate score than the IOCE-low subgroup (Fig. 5C). Furthermore, expression of immune checkpoints including CD274, CTLA4, HAVCR2, TIGIT, LAG3, PDCD1, PDCD1LG2, SIGLEC15 were up-regulated in the IOCE-high subgroup. On the contrary, IOCE-low subtype presented a down-regulation of immune checkpoints (Fig. 5D). As a result, IOCE-high subgroup had an immune-hot phenotype while IOCE-low subgroup had an immune-cold phenotype.

### Construction and validation of the IOCE risk model

**Fig. 6:**
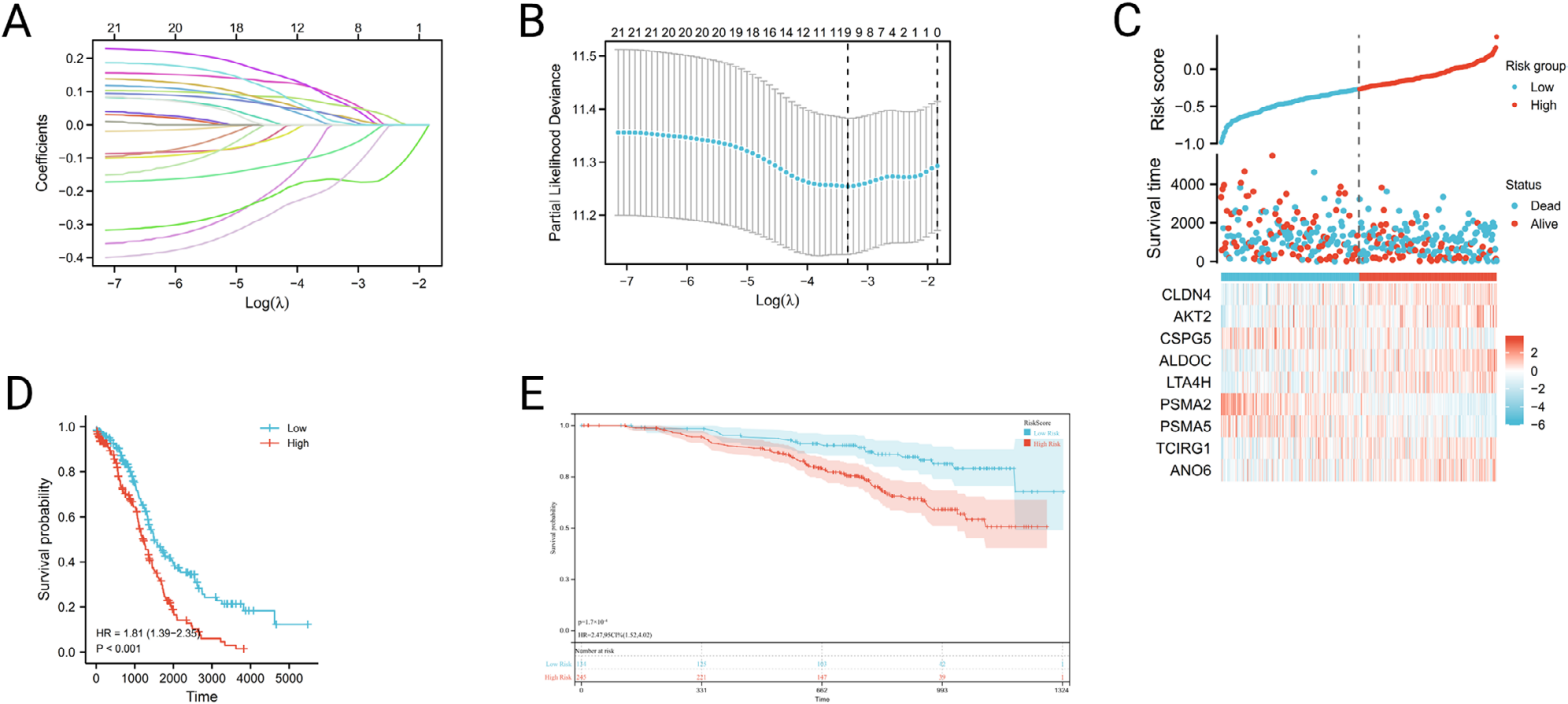
Construction and validation of the IOCE risk model: (**A, B**) Lasso Cox analysis identified 9 genes most associated with OS in TCGA dataset; (**C**) Risk scores distribution, survival status of each patient, and heatmaps of prognostic 9-gene signature in TCGA database; (**D, E**) Kaplan-Meier analyses demonstrate the prognostic significance of the risk model in TCGA and GSE140082 cohort

In order to explore the prognostic value of IOCE in OC patients, a prognostic model based on IOCE-related genes was also constructed. A Lasso regression analysis was performed on 21 IOCE-related genes a to develop a prediction model (Fig. 6A-C). This risk-score model is based on the following algorithm: Riskscore=(-0.0839)×CLDN4+(0.0718)×AKT2+(-0.0762)×CSPG5+(0.0594)×ALDOC+(0.0276)×LTA4H+(-0.1654)×PSMA2+(-0.1723)×PSMA5+(0.0248)×TCIRG1+(0.009)×ANO6. The formula was used to calculate the risk score for each patient, and according to the median value, the patients were grouped into high-risk and low-risk groups. Kaplan-Meier survival analysis revealed a significantly shorter survival rate in the high-risk subtype than in the low-risk subtype (Fig. 6D). Multivariate Cox regression analyses demonstrated that the risk score could serve as an independent prognostic factor in OC (*P* < 0.001, Supplementary Table 4). Furthermore, GSE140082 was downloaded as the validation set to validate the IOCE-related genes risk model established based on TCGA database. Kaplan-Meier survival analysis confirmed that prognosis in the low-risk group was significantly better than that in the high-risk group (*P* = 0.00017; HR = 2.47, 95% CI = 1.52–4.02), which was consistent with the results of TCGA dataset (Fig. 6E).

### Immunohistochemistry verification of 9 IOCE-related genes expression

**Fig. 7:**
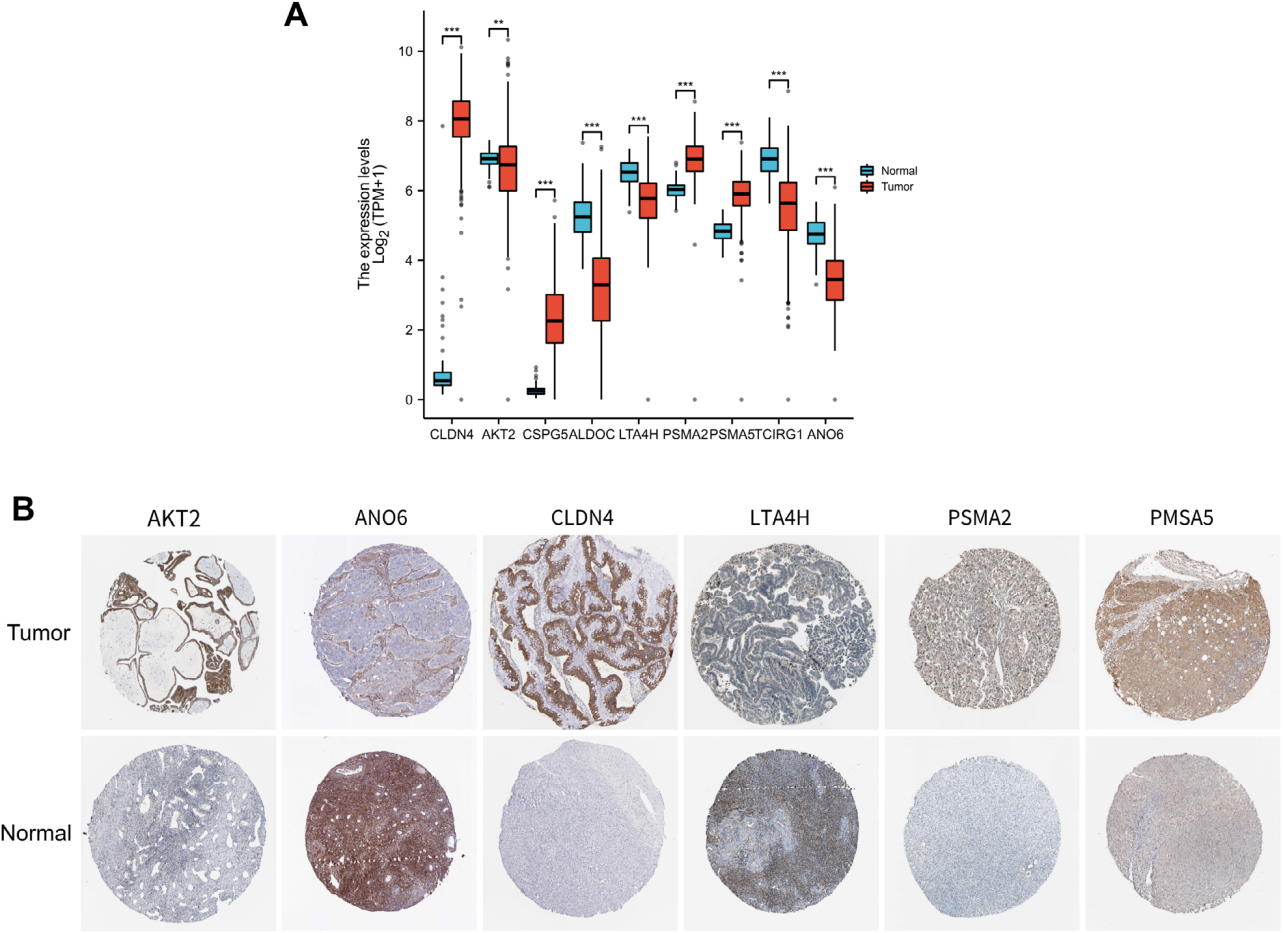
The expression levels of IOCE-related genes in OC tissues and normal tissues: (**A**) mRNA levels of the 9 IOCE-related genes in TCGA; (**B**) Immunohistochemistry verification of IOCE-related genes in OC tissues and normal tissues based on HPA database. Note: *** represents *P* < 0.001

The expression of 9 IOCE-related genes between normal and tumor tissues were compared based on the HPA data. AKT2, CLDN4, PSMA2, and PSMA5, and MSX1, were significantly up-regulated while ANO6 was significantly down-regulated (Fig. 7A-B).

## Discussion

Ovarian cancer (OC) is one of the deadliest cancers of the female reproductive system, which seriously threatens women health. Usually, this cancer has a poor prognosis for its asymptomatic characteristic and high malignancy. Consequently, it is necessary to develop new biomarkers in order to predict this and provide novel targets for treatment. The role of cancer-derived exosomes in OC is complex. There has been evidence that cancer-derived exosomes promote tumor growth and metastasis through their ability to reprogram and instruct other cells [31]. During the process of OC cell dissemination, exosomes from ovarian cancer cells mediated the epithelial-mesenchymal-transition, promote cancer-cell seeding, form metastatic nodules in OC [32, 33]. On the other hand, increasing evidence suggests that the exosome-mediated interaction between cancer cells and the tumor microenvironment plays a crucial role in the peritoneal dissemination of OC [34]. Further, other compartments such as gut microbiota may be a predictor of prognosis and/or response to therapy in sex hormone-related diseases such as in endometriosis, ovarian cancer or polycystic ovary syndrome [35, 36]. This inspired us to explore the potential immunomodulatory properties of exosomes in cancer treatment. The combination of ovarian cancer-derived exosomes and immunity demonstrated multiple roles of exosomes in ovarian cancer and could be beneficial to identify IOCE-associated biomarkers that help predict the prognosis of OC patients and estimate whether they benefit from immunotherapy.

In this study, OC-derived exosomes were harvested from the ExoCarta database and immune-related genes were obtained from the ImmPort database, which were analyzed comprehensively in combination with the TCGA database and GEO database. Here, 21 IOCEs were obtained by an intersection of the DEGs, the exosome-encapsulated genes, immune-related genes and prognosis-related genes. Then, we indicated that the expressions of IOCE genes are closely related to both prognosis and tumor microenvironment of OC. Two IOCE subtypes were identified by consensus clustering based on IOCE-related genes expression. Kaplan-Meier analysis showed IOCE high subgroup presented a poor prognosis but a high level of immune cell infiltration. To identify signal pathways and DEGs involved in different IOCE subgroups, the differential analysis was carried out first, GO and KEGG enrichment analysis were performed. Results indicated the DEGs were involved in ECM-receptor interaction, Focal adhesion, PI3K-Akt signaling pathway, and Cell adhesion molecules (CAMs). GSEA further illustrate the underlying signaling pathways involved in the IOCE high subgroup, and results showed pathways in cancer, gap junction, B cell receptor signaling pathway and T cell receptor signaling pathway were enriched in the IOCE-high subgroup. This is consistent with findings that IOCE high subgroup is associated with a high level of immune cell infiltration.

In addition, we also created a prognosis prediction model with 9 selected IOCE-related genes including CLDN4, AKT2, CSPG5, ALDOC, LTA4H, PSMA2, PSMA5, TCIRG1, and ANO6. For instance, CLDN4 has been reported to be overexpressed and is associated with a poor prognosis in OC [37], which is similar to our results. A down-regulation of CLDN4 makes ovarian cancer cells vulnerable to Taxol and Carboplatin [38]. Another study showed that CLDN4 was involved in angiogenesis, hypomethylation and chemotherapy-resistant in OC [39–41]. Recent study has also demonstrated the expression of CLDN4 could potentially be utilized for assessing immune infiltration in patients with OC [42]. AKT2 plays an important role in immune regulation. It is involved in modulating the function and activity of immune cells, including T cells and macrophages, thereby influencing immune responses and inflammatory reactions [43]. AKT2 expression was increased in primary ovarian carcinomas in comparison with normal ovaries by immunohistochemistry. There was a significant correlation between AKT2 expression and positive lymph node status (*P =* 0.002) and advanced FIGO stage (*P =* 0.009) [44]. Further, AKT2 could promote migration and invasion of ovarian cancer cell by the AKT2-PKM2-STAT3/NF-κB axis [45]. As an extracellular matrix protein encode gene, CSPG5 is associated with the activation of immune cells and inflammatory responses [46]. In addition, CSPG5 is considered a proteoglycan with an EGF-like module that may act as a factor for the growth and differentiation of neurons [47]; ALDOC, belongs to the aldolase family members, plays important roles in glycolysis, fructolysis, and the synthesis of glyceraldehyde and ATP. In several tumor types, ALDOC expression is related to poor clinical outcome [48]. Furthermore, ALDOC is significantly correlated with immune infiltration in gastric cancer by influencing macrophage differentiation [49]. LTA4H is involved in the metabolism of leukotrienes, converting them into biologically active leukotriene B4, which possesses functions in inflammation mediation and immune regulation [50]. In colorectal cancer, LTA4H could be used to evaluate the efficacy of bestatin [51]. In a variety of diseases, PSMA2 has been shown to be diagnostic, prognostic, and therapeutic. For example, PSMA2 enhanced the proliferation, migration and invasion of colorectal cancer [52]. Knockdown of PSMA2 in human lung cells affects the expression of proteins involved in immune and cellular stress responses [53]. According to some studies, PSMA5 was considered to promote the tumour progression of prostate cancer and lung adenocarcinoma while its expression was associated with good prognoses in breast cancer [54–56]. TCIRG1, is recognized as T cell immune regulator 1, constitutes the V0a3 subunit of vacuolar ATPase (V-ATPase), which is involved in varieties of malignant tumors, such as melanoma, breast cancer, and hepatocellular carcinoma [57–59]. ANO6 plays a crucial role in the phosphatidylserine translocation process of the plasma membrane. Interestingly, the inhibition of ANO6 blocks tumor growth by disrupting the delivery of exogenous cholesterol to cancer cells and reversing immune suppression [60]. In glioma, ANO6 induces cancer cell proliferation and invasion by regulating the ERK signaling pathway [61]. Most of the factors have been proved to be associated with immune regulation but not been reported in ovarian cancer, so they may serve as new biomarkers for the disease.

In terms of tumor diagnosis, extracellular-vesicle (EV)-based test has been shown their potential use for early detection in OC [62]. However, there are also literature reports showing that minimally invasive staging is not safer than open techniques or vice versa [57]. On the prognosis prediction of OC, according to the prognosis prediction model with 9 IOCE-related genes including CLDN4, AKT2, CSPG5, ALDOC, LTA4H, PSMA2, PSMA5, TCIRG1, ANO6, we classified the OC patients into high- and low-risk subtypes. Kaplan-Meier analysis demonstrated that high-risk subtype was associated with a dismal clinical outcome. The prognostic model was validated in an external dataset. Overall, this risk model showed a good predictive value in OC and may serve as an independent prognostic indicator for OC patients.

## Conclusions

Our study distinguished two IOCE subtypes by consensus clustering, and the IOCE-high subtype was referred to as immune-hot phenotype, while the IOCE-high subtype was regarded as immune-cold phenotype. Our study revealed the relations of the IOCE subgroups with the tumor immunological microenvironment in OC. These findings may contribute to the development of immune therapy-based interventions for OC patients. Additionally, we developed and validated an IOCE-related prognostic signature that showed significant predictive value for OC survival. Nevertheless, there are certain limitations in our research. First, all ovarian cancer patient cohorts included were retrospective which may cause information bias. Second, since our research results mainly depend on TAGA and GEO database, the reliability of the research needs to be verified by clinical samples due. Due to the complexity of immunoregulation in OC, the exact molecular mechanisms of exosomes in mediating immune response remain to be further explored.

### Electronic supplementary material

Below is the link to the electronic supplementary material.

Supplementary Material 1

Supplementary Material 2

Supplementary Material 3

Supplementary Material 4

## Data Availability

The datasets supporting the conclusions of this article are available in TCGA ovarian cancer dataset (TCGA) (https://portal.gdc.cancer.gov/), GSE140082 dataset (https://www.ncbi.nlm.nih.gov/geo/), the Genotype-Tissue Expression Program (GTEx) (https://commonfund.nih.gov/gtex), the ExoCarta Database (http://www.exocarta.org/), the Gene List module of the Immunology Database and Analysis Portal (ImmPort) (https://www.immport.org/shared/genelists) and the Human Protein Atlas (HPA) (https://www.proteinatlas.org/).

## Abbreviations

CAFs: cancer associated fibroblasts
CAMs: cell adhesion molecules
DEGs: differentially expressed genes
GEO: Gene Expression Omnibus
GO: Gene Ontology
GTEX: Genotype-Tissue Expression Portal
HPA: Human Protein Atlas
IOCEs: immune-related ovarian cancer-derived exosomes
KEGG: Kyoto Encyclopedia of Genes and Genomes
miRNAs: micro RNAs
mRNAs: messenger RNAs
MSigDB: Molecular Signatures Database
OC: ovarian cancer
OCE: ovarian cancer-derived exosomes
OS: overall survival
TCGA: The Cancer Genome Atlas
