# Supplementary material for: An immune-related exosome signature predicts the prognosis and immunotherapy response in ovarian cancer
Source: BMC Womens Health. 2024 Jan 18;24:49. doi: 10.1186/s12905-024-02881-y (PMC10795461; doi:10.1186/s12905-024-02881-y)
Supplement: Supplementary file 4 — Supplementary Material 4 [file 12905_2024_2881_MOESM4_ESM.docx]

| TableS4 Univariate and multivariate cox analysis of Riskscore and overall survival in TCGA-OC cohort | | | | | | |
| --- | --- | --- | --- | --- | --- | --- |
| Characteristics | Total(N) | Univariate analysis | |  | Multivariate analysis | |
|  |  | Hazard ratio (95% CI) | P value |  | Hazard ratio (95% CI) | P value |
| Stage | 303 |  |  |  |  |  |
| I | 1 | Reference |  |  |  |  |
| II | 17 | 0.080 (0.009 - 0.725) | **0.025** |  |  |  |
| III | 236 | 0.176 (0.024 - 1.282) | 0.086 |  |  |  |
| IV | 49 | 0.198 (0.027 - 1.471) | 0.113 |  |  |  |
| Grade | 303 |  |  |  |  |  |
| G1 | 1 | Reference |  |  |  |  |
| G2 | 37 | 138869.9101 (0.000 - Inf) | 0.994 |  |  |  |
| G3 | 264 | 167405.1234 (0.000 - Inf) | 0.994 |  |  |  |
| G4 | 1 | 276683.3113 (0.000 - Inf) | 0.994 |  |  |  |
| Cancer Status | 303 |  |  |  |  |  |
| Tumor free | 80 | Reference |  |  | Reference |  |
| With tumor | 223 | 8.814 (4.635 - 16.762) | **< 0.001** |  | 8.008 (4.185 - 15.324) | **< 0.001** |
| Age | 303 | 1.018 (1.004 - 1.032) | **0.013** |  | 1.016 (1.002 - 1.030) | **0.028** |
| Risk score | 303 | 3.274 (1.766 - 6.072) | **< 0.001** |  | 2.030 (1.032 - 3.992) | **0.040** |
| Anatomic neoplasm subdivision | 303 |  |  |  |  |  |
| Left | 48 | Reference |  |  |  |  |
| Right | 35 | 1.720 (0.975 - 3.035) | 0.061 |  |  |  |
| Bilateral | 220 | 1.267 (0.842 - 1.908) | 0.256 |  |  |  |
